# Keystone Veterinary Medical Association

**Published:** 1892-11

**Authors:** W. S. Kooker

**Affiliations:** Secretary


### SOCIETY PROCEEDINGS.

*Keystone Veterinary Medical Association.*—The regular monthly meeting of the Keystone Veterinary Medical Association was held Sept. 13th, 1892, at the College of Physicians. The meeting was called to order by the President, Dr. W. Horace Hoskins. The minutes of June meeting were read and approved. The Treasurer reported $14.14 in treasury, with $70.00 due from members. The Secretary reported that the attendance of members during the year was up to the average of previous years. There was good work done by the Association, some excellent papers, as well as a great number of cases cited that were instructive. The President made some very pertinent remarks regarding our duty to our Association, to our profession, and to one another They were to the point, and appreciated. Dr. R. G. Webster read a paper on Abortion (*vide* fol. 670, this number of JOURNAL.)

A vote of thanks was tendered to Dr. Webster, for his able paper. There was a lively discussion on the paper, particularly the part relative to the fatty deposits in the uterus.

The President appointed Drs. Bridge, Goentner and Kooker delegates to the United States Veterinary Medical Association, to be held in Boston, commencing September 20th.

The following were elected to serve for the coming year: President, R. G. Webster; Vice-President, J. T. Ferley, Jr.; Secretary, W. S. Kooker; Treasurer, Chas. T. Goentner.

On motion, the election of Trustees was laid over until next meeting. Dr. Hoskins introduced the newly elected officers, with appropriate remarks. Dr. Webster responded, thanking the members for the honor conferred, and hoping each member would do his duty the coming session. On motion, adjourned.

W. S. KOOKER, *Secretary.*

